# Building on Common Ground: Autistic Adults’ and Parents of Young Autistic Children’s Perspectives of Early Behavioral Intervention Practices

**DOI:** 10.3390/bs16040591

**Published:** 2026-04-15

**Authors:** Sophia R. D’Agostino, Naima Bhana-Lopez, Trenton J. Landon, Alyssa Roylance, Avery Briggs

**Affiliations:** Department of Special Education and Rehabilitation Counseling, Utah State University, 2865 Old Main Hill, Logan, UT 84322, USA; naima.bhana@usu.edu (N.B.-L.); trenton.landon@usu.edu (T.J.L.); alyssa.roylance@usu.edu (A.R.); avery.briggs@usu.edu (A.B.)

**Keywords:** early intervention, autistic perspective, parent perspective, ABA, mixed methods

## Abstract

The purpose of this study was to examine the perspectives of autistic adults and parents of autistic children regarding applied behavior analysis (ABA)-based early intervention, their beliefs about best practices in early intervention, and their recommendations for improving service delivery. A mixed-methods design was used to compare quantitative survey data from 70 participants (33 autistic adults, 37 parents) with qualitative interview responses (12 autistic adults, 12 parents), exploring perceptions of ABA and early intervention implementation. Quantitative analyses revealed significant group differences in perceptions of ABA’s benefits and side effects: parents rated ABA-based interventions more positively overall, whereas autistic adults expressed greater concern about potential harms. Despite these differences, both groups endorsed core best-practice principles, emphasizing naturalistic, child-led, and person-centered approaches. Qualitative findings further highlighted the importance of transparency, individualized goal setting, and partnership between families and practitioners. Across groups, participants valued early interventions that promote children’s autonomy and overall well-being. Together, these findings reveal both areas of convergence and divergence in how invested parties perceive ABA-based early intervention and its implementation. Implications for practice include the need to strengthen collaborative, compassionate, and advocacy-driven partnerships among autistic individuals, families, and behavior analysts to enhance social validity, ethical integrity, and inclusivity in early intervention systems.

## 1. Introduction

The importance of early intervention for young autistic children is well documented and supported (Koegel et al., 2014; Sandbank et al., 2020; Sandbank et al., 2023). Applied Behavior Analysis (ABA) is one of the most frequently recommended interventions for young autistic children in the United States (Wong et al., 2015; Vismara & Rogers, 2010). ABA is an evidence-based practice that applies principles of reinforcement and other behavior-analytic procedures to shape behavior and promote skill acquisition (Eckes et al., 2023). ABA-based interventions have shown success in increasing intellectual performance and developmental progress for young children with autism in early intensive behavioral intervention (EIBI; Foxx, 2008; Reichow et al., 2018; Smith et al., 2021).

Foundational ABA studies have emphasized the value of ABA interventions in terms of the ways society (including autistic individuals and parents) benefits from the behavior change achieved by the recipients of the intervention (e.g., Baer et al., 1968, 1987; Wolf, 1978). However, the field of ABA has faced recent scrutiny from the autistic self-advocates who describe ABA-based intervention practices (i.e., discrete trial training) for young children with autism as harmful and aiming to change or cure an individual (Anderson, 2023; Bottema-Beutel et al., 2021). The rise of the neurodiversity movement has increasingly amplified this viewpoint and has led to ethical debates that have impacted autism intervention and research (Leadbitter et al., 2021). On the other hand, parents of autistic children argue that ABA has shown greater acquisition of skills (e.g., language and communicate, social skills, play skills) compared to other interventions and rate it as one of the most important interventions used to help their child (Carlon et al., 2015; Tschida et al., 2021).

Research examining online perceptions of ABA on platforms like Facebook and Reddit confirms this dichotomy in opinions. While many users continuously expressed concerns about ABA being harmful, controversial, or ineffective (Bellon-Harn et al., 2022; Drilling, 2024), some parents advised others to thoroughly research individual clinics, noting that the effectiveness and quality of ABA often depend on the clinic’s practices and the therapist-client relationship (e.g., caseload size, supervision practices, respect for neurodiversity and child autonomy, individualized planning; Drilling, 2024). Reports of abuse and long-term harm from past experiences were countered by comments stating that ABA has evolved, now focusing more on individualized interventions, positive reinforcement, and collaboration with families for intervention planning and caregiver training (Goldstar Rehabilitation, 2025). Overall, these findings emphasize the influential role of social media in shaping perceptions of ABA, particularly among parents and individuals with lived experience and highlight the importance of providing accurate, evidence-based information to guide public perception and treatment choices

Recent guidance on early autism intervention, informed by autistic perspectives and a neurodiversity framework, advocates for moving beyond a sole focus on observable behaviors to include consideration of internal drivers and lived experiences. This approach calls for re-evaluating intervention targets to align with autistic priorities, such as emphasizing strengths and fostering autonomy, and for employing outcome measures that do not center on symptom reduction (Leadbitter et al., 2021). Chazin et al. (2025) conducted an online survey study to recruit autistic adults’ and caregivers’ perspectives on the current, comprehensive range of educational practices commonly used with autistic children. Parents and autistic adults overwhelmingly agreed that the autistic child is the most important stakeholder in decision-making. The study also highlights a clear preference of autistic individuals for affirming, autonomy-supporting practices and parents’ preferences for early intervention environments that adapt to their child’s sensory, emotional, and communication needs rather than forcing conformity, supportive strategies over punitive or behavior-suppressing ones, and active involvement in planning. Further research is essential to ensure that early intervention practices continue evolving in ways that center autistic voices, respect family diversity, and promote meaningful, individualized support to ultimately shape more ethical and effective systems of care.

Best practices in early intervention guide professionals to center caregivers’ priorities and perspectives, particularly during the period immediately following diagnosis, when families are often navigating uncertainty, seeking clarity, and making high-stakes decisions about their child’s care (Bruder, 2010; Crane et al., 2016). Part C of IDEA early intervention guidelines explicitly emphasize the importance of family-centered services, requiring that intervention goals reflect the concerns, routines, and cultural values of caregivers in addition to the developmental needs of the child (Division for Early Childhood, 2014; The Individuals with Disabilities Education Act [IDEA], 2019). Acknowledging caregivers’ priorities at the outset provides a more comprehensive and realistic picture of early intervention decision-making. Integrating both autistic and caregiver perspectives therefore better reflects the complex landscape families encounter and underscores the need for interventions that honor autistic autonomy and family priorities.

As ABA professionals who support this population, our professional responsibility includes ensuring the services we provide do no harm and have positive short- and long-term effects on our clients’ lives (Behavior Analyst Certification Board, 2020). Meeting this responsibility requires listening and learning from all involved parties (e.g., current and past clients, caregivers, professionals) and using their perspectives to inform and improve the services we provide. By including these voices, we can work towards creating interventions and services that are more culturally competent, neurodiversity-affirming, and ethical, while maintaining the highest standards of care for our clients.

When involved parties disagree about intervention approaches and the value of such interventions, as is the case with ABA, the consequences can be significant. On one hand, families may lose access to services that have been shown to support communication and adaptive behavior (Makrygianni et al., 2018). On the other hand, if interventions are experienced as harmful, as reported by many autistic adults (Anderson, 2023; Kirkham, 2017), long-term well-being and trust in services is compromised. Thus, it is key to understand the perspectives of autistic adults and of the parents of young autistic to provide interventions and services that align with the values of both groups and minimize the risk for harm. Yet, there is a gap in the current literature investigating the perspectives of autistic adults and caregivers of autistic children on behavioral interventions.

Although critiques of ABA and calls for reform are increasingly visible, many autistic individuals and autism-community advocates continue to support the availability of early intervention to help young autistic children develop, grow, and thrive (Bent et al., 2024; Leadbitter et al., 2021). However, there remains limited empirical understanding of which aspects of early ABA-based intervention are perceived as acceptable, effective, or harmful by key stakeholder groups, particularly autistic adults and parents of young autistic children. This lack of clarity contributes to polarized and adversarial discourse, which can undermine trust, hinder collaboration, and impede the ethical and effective delivery of services. Autistic adults’ lived experiences provide critical insight into long-term outcomes of early intervention, yet their voices have historically been underrepresented in systematic research informing early intervention practices.

By directly comparing the perspectives of autistic adults and parents, this study addresses a critical gap by identifying areas of convergence and divergence in beliefs about ABA-based early intervention, its implementation, and its communication. Understanding where stakeholder perspectives align versus conflict is essential for distinguishing philosophical disagreement from remediable implementation concerns. Findings from this study have the potential to inform more transparent, respectful, and responsive ABA practices. Ultimately, this work contributes to ongoing efforts to refine early intervention approaches in ways that are developmentally supportive, socially valid, and aligned with the values of those most directly affected. The specific research questions include:

1. How do autistic adults and parents of young autistic children perceive ABA-based early intervention practices, and in what ways do their perspectives converge and diverge?
2. What beliefs do autistic adults and parents of young autistic children hold regarding how early intervention should be implemented, and how do these beliefs compare across groups?
3. What recommendations do autistic adults and parents of young autistic children offer for improving the implementation and communication of ABA-based early intervention?

## 2. Materials and Methods

This study is part of a larger project investigating the experiences of autistic adults and caregivers and draws on the same participant pool and data collection process described in the D’Agostino et al. (2025) study. The research design, recruitment procedures, inclusion criteria, and demographic characteristics are detailed in the earlier publication and are briefly summarized here for context. Questionnaire and interview data were collected as part of a larger study examining the social validity of early intervention practices. While the previous publication (i.e., D’Agostino et al., 2025) explored the social validity and diffusion potential of common naturalistic developmental behavioral intervention strategies implemented in community preschools, the present analysis focuses on different research questions related to adults’ and parents of young autistic children’s perceptions of ABA-based early intervention practices and beliefs about how early intervention should be implemented.

### 2.1. Research Design

For this study, both quantitative and qualitative data were collected and analyzed separately but ultimately integrated to provide a more comprehensive understanding of the research problem. This study used an explanatory sequential mixed method design that explicitly used sequential timing, with the qualitative phase dependent on the quantitative results. The rationale for using an explanatory sequential design is that neither quantitative nor qualitative data alone would sufficiently capture the complexity of autistic adults’ and parents’ perceptions of ABA-based early intervention practices. By combining the breadth of survey data with the depth of interview data, the study seeks to generate findings that are both generalizable and richly contextualized. Quantitative survey data were collected first to identify overall patterns in participants’ perceptions and beliefs toward ABA-based early intervention. The survey included closed-ended items (e.g., Likert-scale ratings) to facilitate statistical comparisons between autistic adults and parents. Following the quantitative phase, qualitative semi-structured interviews were conducted with a subset of participants. The purpose of the interviews was to further explore and elaborate on the quantitative findings, particularly in areas where there were significant differences between groups or where survey responses revealed nuanced or contradictory perspectives.

### 2.2. Inclusion Criteria

Individuals considering participation in the study were initially presented with an informed consent document apprising them of the study parameters and purpose. Individuals who opted to participate were required to answer three inclusion criteria. Both Autistic adults and parents of autistic children were asked to confirm their state of residency. Autistic adults were additionally asked the following questions: (1) Do you self-identify as autistic, have a diagnosis of autism, or are you in the process of receiving a diagnosis of autism?; (2) Are you at least 18 years old and living independently or supported in a way that you can answer the questions on this survey without support workers or family members dictating your responses? Parents of young autistic children were asked the following additional questions: (1) Do you have a child 9 years of age or younger where one of the following applies: (a) Has a formal diagnosis of autism from a medical professional, (b) Is in the process of receiving a diagnosis of autism, (c) Is regarded by a school as having language delay, social communication needs, or social skills needs, (d) Has a medical diagnosis that creates difficulty in speech and/or communication, (e) Currently in the process of determining a diagnosis or school based label reflecting language/communication or social skill deficits?; and (2) Does your child receive/received Applied Behavior Analysis (ABA) based early intervention services?

### 2.3. Participants

Participants in both phases of data collection (questionnaire and interviews) were the same as those reported in the D’Agostino et al. (2025) study. A total of 33 autistic adults and 37 parents of autistic children completed the survey. The group of autistic adults were predominantly female (*n* = 23, 69.7%), White (*n* = 32, 97%) and had some level of post high school education or training (*n* = 23, 69.7%). The mean age was 25.39 years (SD = 7.92). The parental group was also largely female (*n* = 26, 70.3%), white (*n* = 32, 86.5%) and had some level of college or post high school training (*n* = 32, 86.5%). The mean age of parents was 35.3 years (SD = 7.1). At the end of the questionnaire, participants were invited to provide their email address if they were willing to be contacted for a follow-up interview. Twenty-four autistic adults and twenty-five parents of young autistic children opted in by providing their contact information. Of those, 12 autistic adults and 12 parents responded and participated in interviews.

### 2.4. Reflexivity Statement

The research team consisted of five individuals affiliated with a special education and rehabilitation counseling department at a predominantly white institution in the Mountain West U.S. region. All identified as cisgender, with diverse backgrounds in race, disability, and neurodivergence: three authors were white and not disabled, one was white, disabled, and neurodivergent, and one was Latina and Indian with a mental health condition considered neurodivergent by some. Three authors were board-certified behavior analysts (BCBAs). The team acknowledges their professional and personal identities as influential in shaping the research process and interpretation. As a team, we approached the study with varied views of autism, including seeing it as a congenital disability to recognizing it as a different neurotype, but collectively emphasized that societal barriers and expectations are central to the stigma and exclusion autistic individuals face. However, we were unified in promoting inclusion through accommodations, not exclusion, and upheld the belief that participants are experts in their lived experiences. Throughout the research process, we reflected as a team on the fact that traditional ABA practices have caused and may continue to cause harm to many individuals with disabilities. As researchers and clinicians, we are unified in advocating for more natural, neurodiversity-affirming alternatives. Our work is grounded in a commitment to improving supports for autistic individuals by listening to and learning from autistic voices and their support networks. The published reflexivity statement in the D’Agostino et al. (2025) study further elaborates on our neurodiversity-affirming approach and commitment to inclusive, ethically sound practices.

### 2.5. Recruitment and Data Collection

Autistic adults and parents of young autistic children were recruited using purposive sampling through social media, university programs for adults with disabilities, and local parent listservs. Interested individuals accessed an online questionnaire via Qualtrics, provided informed consent, and received a $25 electronic gift card upon completing at least 90% of the survey. Participants could opt in to a follow-up interview by providing their email address at the end of the questionnaire. Those who participated in semi-structured interviews received an additional $25 gift card. All procedures were approved by the university’s institutional review board (#13192).

### 2.6. Measures

The electronic questionnaire was developed using published literature, existing social validity measures, and standard demographic items (e.g., Carter & Wheeler, 2019; Schuck et al., 2022). The questionnaire was piloted with five individuals not included in the study, including two early childhood special education intervention researchers, an autistic adult, a quantitative methods expert, and a parent of a child with a disability. Based on pilot feedback, revisions were made to improve clarity and organization, remove redundant items, and eliminate open-ended response options. Open-ended items were excluded to reduce participant burden and because qualitative data were collected through semi-structured interviews in the subsequent phase of the study.

The current paper focuses on a distinct subset of research questions comprising demographic questions, perceptions of ABA-based early intervention, and beliefs about how early intervention should be implemented questions, which are described below. The demographic questions focused on race/ethnicity, gender identity, employment status, age, median household income, marital status, and level of education. Semi-structured interview questions were created following preliminary analysis of the quantitative data and are described below.

#### 2.6.1. Perception of ABA-Based Early Intervention

Participants were asked to rate their agreement (1 = *strongly disagree* to 6 = *strongly agree*) about seven statements regarding ABA-based early intervention practices. No description or definition of ABA-based early intervention was provided to participants to allow parents and autistic adults to share perspectives on ABA grounded in their experiences. A reliability test for the items specific to ABA-based intervention questions found the Cronbach’s to be 0.82.

#### 2.6.2. Beliefs About How Early Intervention Should Be Implemented

The final section of the survey asked participants to rate their agreement (1 = *strongly disagree*, 2 = *disagree*, 3 = *somewhat disagree*, 4 = *somewhat agree*, 5 = *agree*, and 6 = *strongly agree*) with nine statements regarding beliefs about implementation of early intervention practices. The statements were focused on the context of the intervention setting, intervention planning, use of reinforcers, and the directedness of intervention (e.g., child-led and adult directed) derived from best practice recommendations in early intervention (Division for Early Childhood, 2014; The Individuals with Disabilities Education Act [IDEA], 2019). The early intervention related questions had a Cronbach’s of 0.71. Reliability scores for both questions sets fall within acceptable ranges (Tavakol & Dennick, 2011).

### 2.7. Semi-Structured Interview

The interview guide followed a semi-structured format and was developed after preliminary analysis of the survey data, consistent with an explanatory sequential mixed methods design. Survey findings were used to identify key areas requiring further explanation or contextualization, including patterns of agreement and divergence in perceptions of ABA-based early intervention across participant groups. Interview questions were designed to expand on these findings by eliciting participants’ recommendations for improving ABA-based early intervention, factors informing their perceptions, and their interpretations of why autistic adults and parents may hold similar or differing beliefs. The interview questions were explicitly aligned with the research questions guiding this study and were selected to deepen understanding of the quantitative results. For example, we asked each participant group why they believed their group and the other group’s survey results reflected an overall preference for or against ABA-based early intervention practices. These interview data and questions have not been reported in prior analyses of this dataset.

### 2.8. Data Analysis

Quantitative data analysis was conducted using IBM SPSS Statistics for Windows, version 28 (IBM Corp. (2021), Armonk, NY, USA). Demographic data was summarized using descriptive statistics. Prior to overall data analysis, statistical tests were run to determine if the responses meet assumptions of equal variance. Results for the one-way ANOVA comparing the parent group and autistic adult perspectives of ABA based interventions were statistically significant. Results from the similar one-way ANOVA on the items specific to Early Intervention practices were not significant. Additional analyses using the Welch procedure indicated statistically significant differences on four of the nine early intervention practices.

Additional analyses were conducted to ensure assumptions of normality were met. Visual inspection of boxplots suggested a relatively normal distributional shape with no outliers across both groups on the early intervention practice beliefs. However, the parental perceptions for the ABA-based early intervention perceptions had a slightly positive skew and the autistic adult perceptions had a more negative skew. Despite the skewness reflected by the respective group responses, no significant outliers were noted. To correct for assumptions of normality not being met (Lomax & Hahs-Vaughn, 2012), a series of independent samples *t*-tests were run to compare the perceptions and beliefs of parents of autistic children and autistic adults. A response rate was not calculated as the research team had no way of knowing exactly how many potential participants were invited to participate in this study. Completion rates were noted as follows: 65 parents of autistic children started and 37 completed the parental perceptions questionnaire (56.9%); 53 autistic adults started the questionnaire with 33 completing (62.2%).

Thematic analysis followed the six-phase process outlined by Braun and Clarke (2006, 2019): (1) familiarization with the data and initial coding; (2) collating codes and data extracts; (3) independent theme development and mapping; (4) review and refinement of candidate themes and the thematic map; (5) validation of themes against the full dataset; and (6) production of the final report. In accordance with the initial phases, four members of the research team independently reviewed, coded, and mapped the data. The team then met to evaluate internal coherence within preliminary themes and distinctions between themes, with the goal of establishing consensus on theme labels and definitions that accurately reflected participants’ language. Following consensus, the same researchers reanalyzed the transcripts using the agreed-upon themes and independently developed thematic maps depicting relationships among themes. A final meeting was held to compare maps and reach consensus on the final thematic structure, a process that supported interpretive validity (Altheide & Johnson, 1994). The same analysis procedures were used in the D’Agostino et al. (2025) study.

This study employed a sequential design in which quantitative data were collected and analyzed in the first phase, followed by qualitative data collection and analysis in the second phase. The purposes for mixing data in this study were both developmental and triangulative (Creswell & Plano Clark, 2018; Greene, 2007). Findings from the questionnaire directly informed the development of the semi-structured interview protocol, with specific items and patterns of responses used to generate interview questions. In addition, quantitative and qualitative data were integrated during the interpretation phase to examine participants’ perceptions of early intervention strategies and to identify areas of convergence and divergence across data sources.

## 3. Results

**Figure 1:**
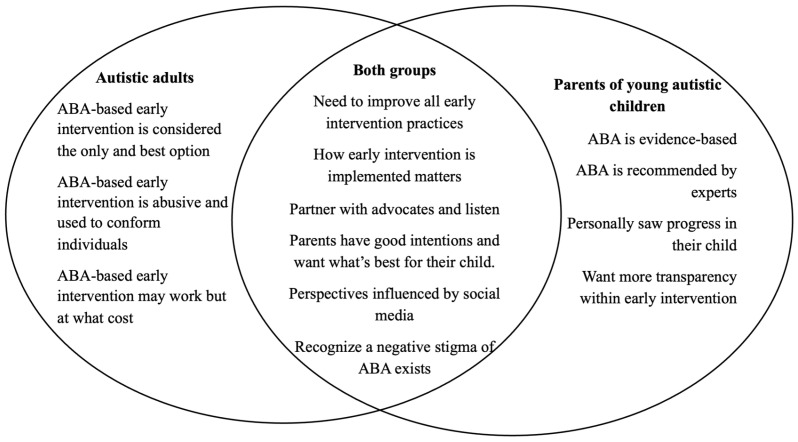
Themes that converge and diverge across participant groups.

This section presents findings that synthesize quantitative and qualitative data to explore parents’ and autistic adults’ perspectives on ABA-based early intervention. The integration of both data strands reveals nuanced patterns of agreement and disagreement regarding early intervention practices. Overall, parents tended to view ABA-based early intervention more favorably, emphasizing perceived effectiveness and meaningful progress in their children’s communication, independence, and behavior. In contrast, autistic adults expressed more critical views, often reflecting firsthand experiences of harm, coercion, or a focus on behavioral conformity rather than support for authentic development. Despite these differing evaluations, both groups shared nuanced understandings of ABA’s complex public reputation and recognized that parents generally approach early intervention decisions with positive intent and trust in professional guidance. Further, both parents and autistic adults emphasized the importance of how early intervention is designed and delivered, while highlighting distinct priorities in what constitutes high-quality, meaningful support. Finally, when asked to provide recommendations for improving ABA-based early intervention, both parents and autistic adults offered insights that emphasized the need for systemic change, stronger collaboration, and more respectful, individualized approaches. These themes are depicted in Figure 1, highlighting areas of convergence and divergence.

### 3.1. Perceptions of ABA-Based Early Intervention Practices

Table 1 presents the quantitative results for the perceptions of ABA-based early intervention practices. Parents consistently reported higher mean ratings of ABA-based early intervention than autistic adults across all positively worded items. Parents rated ABA as highly appropriate (*M* = 5.25; SD = 1.07), effective (*M* = 5.19; SD = 1.02), acceptable (*M* = 5.11; SD = 1.05), and beneficial overall (*M* = 5.30; SD = 1.05), with means generally above 5. Qualitative findings compliment quantitative results. Parent-specific qualitative themes included that parents personally saw progress with ABA-based early intervention and that it is evidence-based and recommended by experts in the field. Interview responses revealed that parents often described personal experiences in which their children made observable progress through ABA-based early intervention. These accounts emphasized perceived gains in communication, daily living skills, and behavioral regulation, which contributed to parents’ generally more favorable views of ABA. One parent shared, “As she went through the program and got the ABA therapy, we saw results. She now speaks, and she’s very competent, and she is very successful in school and in social situations and in almost every situation.” In contrast, autistic adults’ ratings were considerably lower, ranging from 3.48 to 4.00 across these same domains (i.e., appropriateness, effectiveness, acceptability, and beneficial nature of ABA). Autistic adult-specific qualitative themes included acknowledging ABA-based early intervention may work, but at what cost. As one autistic adult participant shared, “I do think it [ABA-based early intervention] is helpful. I do think there are outcomes, and I think it works. I wouldn’t recommend it because a lot of the clinics are not up to date on kindness.”

They also frequently described negative direct experiences with ABA, characterizing the intervention as harmful or abusive. Autistic adult participants shared that they felt self-doubt through their experiences with ABA and experienced coercion. For example, one autistic adult stated:

I actually personally went through ABA. I just think a lot of it felt pretty abusive, and I feel pretty traumatized when I get reminded of it. I will have PTSD flashbacks from that, actually. So, from my experience, it’s a lot of like really negative.

Parents reported stronger agreement with recommending ABA to others (*M* = 5.05; SD = 1.08) and liking ABA procedures (*M* = 4.86; SD = 1.08), compared to autistic adults (*M* = 3.67; SD = 1.81 and *M* = 3.48; SD = 1.60, respectively), with autistic adults expressing greater concern about potential side effects (*M* = 4.21; SD = 1.50) than parents (*M* = 2.73; SD = 1.48). This was reflected in the qualitative data, in which autistic adult participants highlighted concerns that ABA was used to suppress authentic autistic behaviors and enforce conformity to neurotypical norms, rather than supporting individual strengths and autonomy. For example, one autistic adult stated:

Stop trying to make these kids neurotypical. Let’s make sure that the goals that we’re going to address are going to be so that they have a better quality of life to communicate, but not to totally stop behaviors that are seen as inconvenient or weird.

Despite differences in perspectives of ABA-based early intervention practices, qualitative findings revealed areas of agreement. First, a theme from both parents and autistic adults was the acknowledgement that ABA carries a negative public reputation. Both participant groups also noted that much of their perspectives are influenced by social media, where critical perspectives are frequently shared and debated. An autistic adult explained:

Because a lot of what we know nowadays comes from content creators on like TikTok and Instagram and the platforms like Youtube and stuff. Because that’s where we get our information from is the people we trust and we can relate to online.

Similarly, a parent shared, “during my trying to figure out what the heck autism is, it was a lot of social media information.” Further, both parents and autistic adults emphasized that parents generally want what is best for their children and approach early intervention decision-making with positive intentions. One parent shared, “And as a parent who just wants my child to be able to function in the world that he lives in, I want to help him have a meaningful life.” Similarly, an autistic adult said:

They [parents] always have the best of intentions, and what they’re seeing is a child who is not able to be, I hate to use the word normal, but you know, “normal”. And in the parents’ minds that’s going to reduce bullying, they’re going to be able to make friends, they might be able to stay on track with school, all of these good things.

Another theme from autistic adult participants was that they often observed that parents view ABA as the only or best available intervention option. From their perspective, this reliance reflects not just trust in evidence but also systemic pressures that position ABA as the default choice in early intervention. Similarly, parents frequently described ABA as the only evidence-based practice endorsed by professionals and service systems. This external validation contributed to their trust in ABA as an appropriate intervention for their child while acknowledging the perspectives of autistic adults. One parent shared:

I’ve read the perspectives like, “We don’t need to be changed. We’re who we are, and why do we need to change?” But at the same time, watching my little girl before she had the ABA, her quality of life was not awesome.

### 3.2. Beliefs About Early Intervention Implementation

Table 2 presents the quantitative results for the early intervention beliefs statements. Overall, both parents and autistic adults expressed strong endorsement of best-practice principles in early intervention. Both groups rated highly the importance of person-centered planning (Parents *M* = 5.41; SD = 0.96; Autistic Adults *M* = 5.36; SD = 0.70), consideration of developmental sequences (*M* = 5.38 vs. 5.18), and the use of natural, child-preferred reinforcers (*M* = 5.22; SD = 1.03 vs. 5.06; SD = 1.09). Similarly, both groups disagreed that standardized assessments alone should guide goals, with means closer to the lower end of the scale (Parents *M* = 2.70; SD = 1.41; Autistic Adults *M* = 3.00; SD = 1.73), and neither group strongly supported interventions being mostly adult-directed (Parents *M* = 3.32; SD = 1.42; Autistic Adults *M* = 3.18; SD = 1.59).

Although both participant groups agreed, the degree of emphasis placed on child-led and naturalistic approaches differed. Autistic adults rated higher the importance of delivering intervention in natural environments (*M* = 5.33 vs. 4.92), tailoring intervention to the child’s interests and everyday life (*M* = 5.45 vs. 5.00) and supporting child-led approaches (*M* = 5.12 vs. 4.35). Further, parents reported slightly higher endorsement of allowing reinforcers unconnected to the activity (*M* = 3.86 vs. 3.21).

One qualitative theme revealed that both groups agreed that the effectiveness and acceptability of early intervention depend heavily on how it is implemented. Participants stressed that intervention is not inherently positive or negative; rather, outcomes are shaped by the manner in which strategies are applied, the responsiveness of providers, and the degree of alignment with the child’s and family’s needs. One autistic adult participant explained, “And so I can also see how when ABA is done with, when it’s done with empathy and care when they’re trying as hard as they can to do it right, it’s not necessarily like an evil thing.”

### 3.3. Recommendations for Improving Delivery of ABA-Based Early Intervention

Both groups noted that their concerns and suggestions extended beyond ABA to early intervention practices in general. Participants emphasized that improvements to transparency, collaboration, and responsiveness are needed across the full spectrum of services available to young autistic children and their families. Parents and autistic adults alike stressed the importance of listening more carefully to autistic voices, parent experiences, and advocacy communities. They suggested that incorporating multiple perspectives would ensure that interventions are not only evidence-informed but also socially valid and responsive to those directly affected. One autistic adult participant explained:

It may take us a while for us to build that trust because it takes us a while to build trust sometimes, after being burned so many, many times. We can do it, and we want to be involved. That is the biggest thing.

Similarly, a parent participant recommended, “So ask the adults with autism, “Well, what would you change?” or “How do we make this better?” and letting them manipulate a little bit.”

A theme from the Autistic adult participants emphasized that early intervention should prioritize outcomes that are meaningful to the autistic individual rather than narrowly focused on compliance or normalization. They advocated for individualized, client-driven goals that honor the child’s autonomy, preferences, and long-term quality of life. One autistic adult participant emphasized:

But I don’t think the goal of these therapies should be to, “Oh, they’re just normal, and everything’s fine.” Let’s not do that. Let’s make sure the goals are to address a quality of life so they can learn and stuff like that, but not take away from who they are.

A theme from parent participant responses also highlighted the need for more collaborative partnerships with clinicians, in which families are treated as active and respected members of the intervention team and underscored the importance of the therapeutic relationship between providers and families. They emphasized that clinicians should build trust, communicate openly, and demonstrate sensitivity to family contexts and values. Parents also expressed a strong need for transparency in early intervention. They wanted clear communication from providers regarding methods, goals, and rationales so they could better understand and evaluate the interventions their children were receiving. Transparency was viewed as essential for building trust and confidence in the intervention process. One parent stated, “So I would highly recommend transparency with parents, and just let them know what’s going on. Keep them involved.”

## 4. Discussion

This study aimed to investigate the perspectives of autistic adults and parents of children on the autism spectrum regarding ABA-based intervention, their beliefs about early intervention practices, and their recommendation for improving the delivery of these services. Across both sets of items, autistic adults expressed views that revealed both common ground and points of divergence regarding perspectives of ABA-based early intervention and beliefs about early intervention practices. Our findings indicate that participant groups held some similar perceptions and some significantly different perceptions of ABA-based practices.

In general, autistic adults had lower opinions about the benefits of ABA-based services, and on average, only slightly agreed that ABA was beneficial to young children with autism. These results indicated a more positive opinion of ABA than previous research (Kirkham, 2017). On the other hand, parents of autistic children tended to agree or strongly agree that ABA was beneficial to young children with autism and generally liked the procedures used in traditional ABA-based services, which aligns with parents’ opinions discussed in previous research (Tschida et al., 2021). These differences may reflect the distinct vantage points from which each group interacts with ABA. Parents typically experience ABA as caregivers observing their child’s progress, whereas autistic adults draw on their lived experiences as former recipients of services. Differences in goals, informational sources, developmental timing, and the evolution of ABA practices over time may further contribute to these divergent views. Although both groups tended to agree that ABA procedures were effective in increasing the skills of young children with autism, they disagreed on whether they resulted in negative side effects for those children. Autistic adults, on average, agreed that negative side effects were a result of ABA-based procedures, while parents typically disagreed. These results align with the current understanding of autistic adults’ and parents’ opinions of side effects of ABA (Carlon et al., 2015, Anderson, 2023).

The findings regarding early intervention practice beliefs suggest strong areas of alignment between parents and autistic adults regarding the foundational principles of early intervention, alongside some variation in the extent to which each group prioritizes naturalistic and child-led approaches. Both participant groups within our study believed that early intervention practices should take place in the child’s natural environment(s), intervention planning should focus on the child’s interests, goals should be developed with person-centered planning and consider developmental norms, reinforcers should be natural, and the intervention should be mostly child-led.

In a recent study, Chazin et al. (2025) surveyed 226 autistic individuals, and their items extrapolated more nuance regarding the social validity of general autism early intervention practices. In alignment with our results, they found that autistic adults view some ABA-based procedures more favorably than others. In contrast, their findings emphasize that the learning context should be individualized, which may not be tied to the child’s natural environment. Our findings suggest that while parents generally hold more favorable perceptions of ABA as an intervention approach, both groups align on key best-practice principles in early intervention. Autistic adults, however, place relatively greater emphasis on naturalistic, child-led approaches and express heightened concern about possible negative side effects, highlighting areas where priorities and experiences may diverge. Overall, both studies point to the importance of autonomy within autism early intervention practices.

Naturalistic and child-led approaches align well with Naturalistic Developmental Behavioral Intervention (NDBI), which both autistic adults and parents of young autistic children tend to regard as more socially valid (D’Agostino et al., 2025). In addition, both groups emphasized the importance of partnering with advocates and listening to diverse perspectives to ensure that practices are responsive, inclusive, and ethical. This finding is in line with recent research in the field, that calls for behaviorists to reflect, listen, and include autistic advocates, collaborate with related service professionals, as well as culturally and linguistically diverse invested parties (e.g., caregivers, educators, therapists) to improve their provision of services (Kirby et al., 2022; Mathur et al., 2024). Parents additionally identified specific areas for improvement in collaboration, reinforcement strategies, and clinician relationships, highlighting the practical dimensions of intervention delivery that directly shape family experiences. These findings underscore the need for early intervention systems to balance effectiveness with transparency, respect, and social validity.

### 4.1. The Role of Social Perception

Providers of socially valid ABA services are mindful of the following questions: (1) Is what we are doing what society wants? (2) Is the procedure we are developing acceptable to everyone? and (3) Is everyone happy with the results? (Prchal, 2020). Such an orientation focused on providing a desirable good or service is like the concept of customer satisfaction in the business and marketing world. Customer satisfaction has been found in business sectors to impact “word of mouth” referral to new customers (Wangenheim & Bayón, 2007). When a good or service is considered worthwhile, consumers of the good or service will recommend or refer the service to their friends and acquaintances within their social network based on a positive evaluation of the good or service.

Like other consumer goods or services (Xevelonakis, 2016), those opinions and subsequent recommendations (either positive or negative) by the autistic community and other invested parties have the potential to influence public perception. An individual’s social network has been shown to have a greater impact on the proliferation of a good or service than any other source (Xevelonakis, 2016), therefore a positive or negative appraisal of ABA-based intervention practices by a friend or family member is likely to have a strong impact on an individuals’ overall perception of ABA-based practices. Moreover, the perceived relationship between the provider of the good/service and the consumer impacts the consumer’s willingness to share referrals with the provider (Johnson et al., 2003). When considering the recent viewpoints of many autism advocates and professionals that ABA is unethical and inhumane (Wilkenfeld & McCarthy, 2020), this would conceivably have an adverse impact on word-of-mouth referrals for ABA-based services within the autism community and the social networks of autistic individuals. Our findings align with this perspective, indicating that perceptions of ABA may indeed influence willingness to recommend or engage with such services. The perceptions of the intervention, regardless of clinical significance, may be impacted based on a negative social perception of the intervention. Hahn (2025) surveyed parents, practitioners, and teachers using TikTok content and found that these social media posts did not change participants’ opinions of ABA. However, participants believed such content might influence others, which underscores a perception gap between potential influence on others versus the self.

If a strategy for behavior change is misunderstood by the targeted intervention group or supporting individuals regardless of its potential use and evidentiary support, accessing the intervention and fidelity to the strategy are likely to be compromised. Therefore, the final selection of an intervention may be based on parental values, perceived child need, and accessibility to the intervention (Saez et al., 2023). Our findings reinforce this point: perceptions of ABA within the autism community, particularly skepticism among autistic adults, may limit access to or sustained engagement with ABA-based interventions. These findings emphasize the importance of transparency, collaboration, and alignment between intervention practices and the values of families and the broader community. However, our results also highlight areas of convergence: both groups valued interventions that enhance children’s independence, communication, and overall well-being. Recognizing this common ground presents an opportunity to bridge perspectives and promote collaboration in designing early interventions that reflect shared priorities.

### 4.2. Implications for Practice: Building on Common Ground

#### Supporting Collaborative Relationships

When forging collaborative relationships between the behavior analytic community, autistic individuals, and relevant invested parties, like parents, building on common ground to develop shared visions and goals is essential for effective collaboration (Foster-Fishman et al., 2001), ultimately leading to more socially valid interventions. Findings from this study highlight several areas of alignment between autistic adults and parents of young autistic children that can serve as a foundation for consensus in shaping ABA and early intervention practices. While autistic adult participants in this study emphasized that ABA could be and had been harmful from their perspective, both participant groups viewed ABA as effective in changing behavior and agreed on numerous aspects of how early intervention should be implemented. Moreover, interviews with participants from both groups revealed empathetic perspective–taking (i.e., and effort to understand one another’s experiences and viewpoints). However, participants also differed on key issues: autistic adults expressed greater concern about potential side effects of ABA services.

To strengthen collaborative partnerships, it is essential to cultivate mutual respect and trust among all involved parties. This respect and awareness of diverse perspectives are also key principles of cultural competence, another important skill for behavior analysts to develop (Fong & Tanaka, 2013). As future steps for early intervention are contemplated, thoughtful consideration should be given to both the points of convergence and divergence between stakeholder groups when making decisions about the individuals we serve across all severity levels. These collaborations directly align with the BACB Ethics Code for Behavior Analysts (Behavior Analyst Certification Board, 2020), which emphasizes the importance of engaging relevant parties in the treatment plan and of seeking input to ensure that interventions respect the dignity of our clients and promote meaningful, socially valid, and culturally responsive goals and outcomes. Recent initiatives in which critical autism scholars and behavior analysts come together to “speak across the divide” and discuss how ABA can evolve exemplify this progress and demonstrate a promising step toward more collaborative and reflexive practices (Jackson-Perry et al., 2025; Suckle et al., 2025)

### 4.3. Emphasizing Compassionate Care in ABA

The field of ABA is increasingly recognizing the importance of compassionate care as an essential component of ethical and effective practice (D’Agostino et al., 2023; LeBlanc et al., 2020; Taylor et al., 2019). In recent years, the field has produced a growing number of intervention studies focused on soft skills training as a way to increase compassionate care within behavior-analytic practice. For instance, a systematic review by Barrera-Lansford et al. (in press) identified eleven studies that provided soft-skills training to behavior specialists and/or graduate students in the field. However, despite emerging literature and intervention studies in this area, the field of ABA has not explicitly partnered with autistic individuals to gather their perspectives. Centering autistic voices involves prioritizing the input of clients in treatment planning, while still valuing the expertise of clinicians and the perspectives of family members (Mathur et al., 2024). This approach is critical because it fosters ethical, person-centered practice and enhances the likelihood that interventions will be meaningful and creates a foundation of shared understanding among all parties, thereby building common ground for collaboration.

#### Promoting Shared Advocacy

Developing unified advocacy statements that are supported by behavior analysts, parents, and autistic individuals can provide a shared framework for guiding early intervention practices. These statements can clarify shared values and principles, creating a foundation for collaboration. Disagreement is natural and expected. Focusing on areas of common ground, rather than complete consensus, allows progress in intervention design and decision-making while respecting diverse perspectives. Evanko et al. (2025) note that collective action requires identifying areas of agreement rather than uniformity of opinion; meaningful progress in policy and practice depends on a shared understanding of what behavior analysis is and what it can offer. Advocacy efforts are strengthened when multiple groups come together, acknowledging that no single group has all the answers. The development and adoption of such a unified statement could promote more collaborative partnerships among practitioners, caregivers, and the autistic community, ultimately leading to more compassionate, ethical, individualized, and effective early intervention practices. Collaborative efforts can guide policy, research, and practice in ways that are inclusive, responsive, and aligned with the values of the autism community.

### 4.4. Limitations and Future Research Directions

This mixed methods study highlights areas of common ground among autistic adults and parents of young autistic children regarding early intervention practices. However, findings should be considered in light of several limitations. First, this study’s sample of 70 total survey participants (33 autistic adults, 37 parents) limits the generalizability of findings. Both groups were predominantly white, female, and highly educated, which may not reflect the full diversity of families and autistic adults across racial, cultural, or socioeconomic contexts. Recruitment through social media and university networks may have introduced selection bias, favoring individuals already engaged in autism discourse or advocacy. Future studies should intentionally recruit more racially, ethnically, and socioeconomically diverse participants, as well as fathers, gender-diverse individuals, and non-English speakers, to examine how intersecting identities shape perceptions of early intervention. Also, a limitation of our inclusion criterion for autistic adults is that it excluded autistic adults who rely on support workers or family members to assist with communication or decision-making, resulting in a sample that may overrepresent individuals with higher levels of independence. Consequently, the findings may not fully reflect the perspectives of autistic adults with greater support needs, limiting the generalizability of the results across the full spectrum of autism. Future research could incorporate adapted communication methods or caregiver-assisted reporting with safeguards to effectively incorporate the perspectives of autistic individuals who require higher levels of support. Next, both survey and interview data rely on self-reported perceptions, which may be influenced by participants past experiences, recall bias, or social desirability. For autistic adults, retrospective accounts and perspectives of early intervention may have been influenced by current advocacy perspectives or exposure to online discourse. Also, we did not collect data regarding the prior service history of autistic adults or autistic children. Future research could combine perception data with direct observation of intervention delivery or outcome measures to allow triangulation between reported beliefs and actual practices or child outcomes. Finally, this study intentionally did not define “ABA-based early intervention”, which allowed participants to respond based on their own understanding. Differences in interpretation could have affected how participants rated their perceptions, especially given the wide variation in how ABA is practiced across settings. Future research could provide standardized definitions or descriptions of intervention types to examine whether perceptions differ based on exposure to traditional versus naturalistic or neurodiversity-affirming ABA practices.

## 5. Conclusions

These findings illustrate both areas of convergence and divergence in perspectives regarding perception of ABA beliefs about early intervention service implementation. Shared recognition of ABA’s stigma and of parents’ good intentions suggests a foundation of common understanding. However, parents’ favorable views, shaped by observed child progress and professional endorsement, contrast with autistic adults’ critical evaluations, which emphasize lived experiences of harm, systemic pressures, and the ethical costs of intervention. These differences help explain the quantitative results in which parents rated ABA more positively overall, while autistic adults reported greater concern about negative side effects. Similarly, both groups agreed that early intervention implementation quality is central, underscoring the importance of provider skill and intervention design. Parents prioritized transparency to participate as informed partners in their child’s intervention, while autistic adults stressed the importance of ensuring that intervention goals are individualized and driven by the child’s needs and values. Together, these perspectives suggest that early intervention may be most effective when it is both transparent for families and authentically centered on the autistic individual’s autonomy and well-being. Overall, these findings underscore the importance of advancing early intervention through collaborative, compassionate, and advocacy-driven partnerships that bridge diverse perspectives and promote socially valid, person-centered care.

## Figures and Tables

**Table 1:** Perceptions of ABA-based Early Intervention.

Prompt	Parent Group (*n* = 37)		Autistic Adults (*n* = 33)		t Score	df	Cohen’s d
	*M*	SD	*M*	SD			
I find ABA-based early intervention appropriate for young children with autism.	5.25	1.07	3.61	1.75	4.79 *	51.64 ^a^	1.43
ABA-based early intervention is effective in increasing the skills of young children with autism.	5.19	1.02	4.0	1.74	3.59 *	50.99 ^a^	1.39
I would suggest the use of ABA-based early intervention to others.	5.05	1.08	3.67	1.81	3.95 *	50.87 ^a^	1.47
ABA-based early interventions do result in negative side-effects for children with autism.	2.73	1.48	4.21	1.50	−4.16 *	68	1.49
ABA-based early intervention is an acceptable way to target the child’s skills.	5.11	1.05	3.79	1.60	4.13 *	54.23 ^a^	1.33
I like the procedures used in ABA-based early intervention.	4.86	1.08	3.48	1.60	4.26 *	55.26 ^a^	1.35
Overall, ABA-based intervention is beneficial to young children with autism.	5.30	1.05	3.82	1.70	4.36 *	51.36 ^a^	1.42

* *p* ≤ 0.001; ^a^ Equal variance not assumed.

**Table 2:** Early Intervention Practice Beliefs.

Prompt	Parents (*n* = 37)		Autistic Adults (*n* = 33)		t Score	df	Cohen’s d
	*M*	SD	*M*	SD			
The intervention should take place in the child’s natural environment(s).	4.92	1.09	5.33	0.74	−1.84 *	68	0.94
The child’s interests and everyday life should determine the intervention setting and materials.	5.00	1.12	5.45	0.62	−2.09 *	68	0.91
The developmental sequence should be considered when developing intervention goals.	5.38	0.92	5.18	0.73	0.98	68	0.84
Person-centered planning should be used to develop intervention goals.	5.41	0.96	5.36	0.70	0.21	68	0.85
Standardized assessments alone should be used to guide intervention goals.	2.70	1.41	3.00	1.73	−0.78	61.85 ^a^	1.57
The reinforcers can be unconnected to the activity and chosen provider.	3.86	1.44	3.21	1.76	1.71 *	68	1.60
The reinforcers should be natural depending on the child’s preference and the activity.	5.22	1.03	5.06	1.09	0.61	68	1.06
The intervention should be mostly adult directed.	3.32	1.42	3.18	1.59	0.39	68	1.5
The intervention should be mostly child-led. **	4.35	1.30	5.12	0.99	−2.81 **	66.55 ^a^	1.16

* *p* ≤ 0.05; ** *p* ≤ 0.005; ^a^ Equal variance not assumed.

## Data Availability

The data presented in this study are available on request from the corresponding author due to ethical restrictions.

## Abbreviations

The following abbreviations are used in this manuscript:

ABA	Applied Behavior Analysis
NDBI	Naturalistic Developmental Behavioral Intervention
